# The Treatment of Consumption

**Published:** 1880-10

**Authors:** 


### THE TREATMENT OF CONSUMPTION.

In a paper on the treatment of pulmonary consumption, Prof. Péter, of Paris, insists strongly on the value of hydrotherapy. He begins with frictions with dry flannel, then passes to rubbing with cloths dipped in aromatic alcohol, cologne water, or vinegar, followed by dry friction for five or six minutes, and finally advances to the use of the cold sponge. The process is repeated twice daily, immediately after rising and before retiring. He believes sponging to be better than the douche, because it is more easily carried out. The chief points to be observed are, to accustom the patient gradually to the use of cold water, and not to prolong the bath too much at first. Prof. Péter divides the sweats of phthisis into three classes, according to their cause, viz.: ordinary night-sweats, which depend not so much on the pulmonary trouble as on the general condition and the tubercular fever, the sweating which follows high evenings exacerbations of the fever, and colliquative sweats. To control the first, he recommends especially sponging with vinegar, combined with the usual internal remedies, such as acetate of lead, tannin, etc. Atropine, he considers unreliable. Quinine is useful for the second form, because it controls the fever. For the colliquative sweats, there is no remedy. For the cough, he gives opium and belladonna in small doses; he orders pills containing one-sixth of a grain of opium, and one-twelfth of a grain of ext. belladonna, and gives, at first, one at a dose, increasing afterward if necessary. When the cough causes vomiting, he gives one or two drops of tincture of opium before meals, with good effects. When the vomiting seems to be due more to dyspepsia than to the cough, he gives a few drops of hydrochloric acid after the meals. In such cases, alcohol in some form is also useful, but it must be given freely. For the diarrhœa, when it is due to simple intestinal catarrh, as is usually the case at the outset of the disease, he employs subnitrate of bismuth, in connection with a carefully regulated diet. When it is due to the use of cod-liver oil, or to the milk or grape cure, the exciting cause must be discontinued, and the stomach, if overloaded, be emptied by an emetic. When it is due to inflammation of the stomach and intestines, he prescribes opium, nitrate of silver, perchloride of iron, etc., and employs also derivatives to the skin. For colliquative diarrhœa there is no remedy. For controlling the expectorations, he has found the balsams, glycerine, and kermes, to be the best remedies. For hæmoptysis, he recommends in the first place, the use of emetics, and explains their action on the theory that they excite a reflex action through the sympathetic, which causes anæmia of the lungs, and controls the hemorrhage. When patients have been greatly reduced by the hæmoptysis, he has found quinine and ergotine useful.—*Allg. med. Cent. Zeit.*, February 25, 1880.—*Med. Record.*

